# A poor prognosis in human hepatocellular carcinoma is associated with low expression of DPP4

**DOI:** 10.1590/1414-431X20209114

**Published:** 2020-04-09

**Authors:** Hao Yu, Xiao-Ping Mei, Peng-Fei Su, Guang-Zhi Jin, Hong-Kun Zhou

**Affiliations:** 1 Department of Hepatobiliary Surgery, The First Hospital of Jiaxing, Jiaxing, Zhejiang, China; 2 Department of Hepatobiliary Surgery, The First Affiliated Hospital of Jiaxing College, Jiaxing, Zhejiang, China; 3 Department of General Surgery, Central Hospital of Liaoyang, Liaoyang, Liaoning, China; 4 Department of Pathology, Eastern Hepatobiliary Surgery Hospital, The Second Military Medical University, Shanghai, China

**Keywords:** Dipeptidyl peptidase 4, Hepatocellular carcinoma, Immunohistochemistry, X-tile, Overall survival, Patient-derived xenograft

## Abstract

This study aimed to explore the prognostic role of dipeptidyl peptidase 4 (DPP4) expression in hepatocellular carcinoma (HCC). DPP4 expression was measured in formalin-fixed paraffin-embedded specimens that were gathered from 327 HCC patients. Immunohistochemistry analyses were utilized to examine DPP4 expression characteristics and prognostic values (overall survival (OS) and time to recurrence) of DDP4 in HCC tissues. In addition, a patient-derived xenograft (PDX) model was used to assess the correlation between DPP4 expression and tumor growth *in vivo*. DPP4 was expressed in low levels in HCC tissues in contrast to paired peritumoral tissues (38 cases were down-regulated in a total of 59 cases, 64.4%. P=0.0202). DPP4 expression was significantly correlated with TNM stage (P=0.038), tumor number (P=0.035), and vascular invasion (P=0.024), and significantly reduced in patients who were in TNM stages II and III-V, with multiple tumors, and with microvascular invasion compared to patients with TNM stage I, single tumor, and no microvascular invasion. Notably, HCC tissues with low expression of DPP4 had poor OS (P=0.016) compared with HCC tissues with high expression of DPP4, and results from PDX model showed that tumor growth was significantly faster in HCC patients that lowly expressed DPP4 compared to those with highly expressed DPP4. Our findings suggested that low levels of DPP4 could impact the aggressiveness of HCC and contribute to a poor prognosis.

## Introduction

Hepatocellular carcinoma (HCC) is one of the most frequently diagnosed cancers across the globe. It accounts for about 50% of the overall number of cancer cases as well as deaths in China (1,2). The 5-year recurrence rate remains at 70–80% even though hepatectomy has been used as a routine therapeutic regimen to improve the survival rate of HCC patients (3,4). Developing a novel useful biomarker to distinguish HCC patients with high or low risk for survival or recurrence could provide guidance for clinicians when selecting rational therapeutic strategies and adopting personalized therapies (5–7).

Dipeptidyl peptidase-4 (DPP4), an ubiquitously expressed transmembrane glycoprotein, is a possible marker for many cancers. However, there is variability between various forms of cancers regarding the efficacy of DPP4 (8). DPP4 is upregulated in many aggressive types of T-cell malignancies (9), esophageal adenocarcinoma (10), lung adenocarcinoma (11), thyroid carcinoma (12), and prostate cancer (13), and a high level of DPP4 is indicative of poor prognosis for papillary thyroid carcinoma (14), urothelial carcinoma (15), and colorectal cancer (16). In contrast, DPP4 is downregulated in tissues of liver cirrhosis (17), endometrial adenocarcinoma tissues (18), and a low serum level of DPP4 was related to a poor prognosis for patients who have esophageal squamous cell carcinoma (19).

The expression of DPP4 in HCC tissues and the impact on the prognosis for HCC patients remains unknown. To answer this question, we assessed DPP4 expression characteristics in HCC and peritumoral liver tissues using immunohistochemistry. We also investigated the relationship between DPP4 expression levels and the prognostic value of these levels in HCC tissues. A patient-derived xenograft (PDX) model was utilized to examine the correlation among DPP4 expression and tumor growth *in vivo*.

## Material and Methods

### Patient tissue samples and follow-up

Formalin-fixed paraffin-embedded (FFPE) samples (a total of 327) were selected randomly. Among these, 59 cases of HCC with paired peritumoral tissues were employed as an expression pattern cohort. These samples were obtained from The First Hospital of Jiaxing from January 2016 to December 2017. As the prognosis cohort, 268 HCC cases without paired peritumoral tissues were recruited at the Eastern Hepatobiliary Surgery Hospital between January 2006 and December 2012. Follow-up continued until December 2016. Hematoxylin and eosin (HE)-stained sections from every FFPE tissue were fixed on slides and reviewed by two experienced pathologists.

The enrollment criteria for patients were: 1) diagnosis of HCC based on the criteria proposed by the World Health Organization (WHO); 2) pathological diagnosis of HCC; 3) no pre-operative anti-cancer treatment; 4) no extrahepatic metastases; and 5) complete follow-up data ready for the prognosis cohort (268 cases). Written informed consent from all of the subjects was obtained and Institutional Review Board approval was granted (EHBHKY2014-03-006 by ENBH and LS2019-038 by The First Hospital of Jiaxing).

The overall survival (OS) was specified as the period of time between surgery and death or surgery and the last observation. The time-to-recurrence was established as the time between the date of tumor resection up to the detection of tumor recurrence, the last observation, or death. Patient follow-up occurred every 3 months throughout the first 12 months after surgery, and then follow-up visits occurred every 6 months until December 2016. Follow-up examinations were conducted by 2 physicians who were unaware of the study. All of the patients were monitored using chest X-ray, abdomen ultrasonography, and serum alpha fetoprotein (AFP) concentrations during 1-6 months after the surgery and every 3-6 months after that. Scanning or magnetic resonance imaging and computed tomography of the abdomen were performed every 6 months or immediately after a suspected recurrence. The diagnosis criteria for recurrence were the same as that for the preoperative diagnosis (5).

### Tissue microarray, immunohistochemistry, and scoring

Immunohistochemistry, tissue microarray construction, and calculation of protein expression levels were performed as previously reported (20–22).

In brief, HE-stained slides were evaluated by pathologists and the representative cores were pre-marked in the paraffin blocks. A cylinder of tissue with a diameter of 1.0 mm was removed from a designated portion of each block. This cylinder was then placed into a recipient paraffin block. Sections that were 4-μm thick were then put on slides that were coated with 3-aminopropyltriethoxysilane. Sections were deparaffinized in xylene (2 times, 10 min each time) and rehydrated using 100, 95, and 85% ethanol for 5 min each time. Microwave irradiation for 5 min in citric buffer (pH 6.0) was performed to unmask antigens, which were then cooled for 120 min at room temperature.

The slides were incubated in phosphate-buffered saline (PBS) in 3% H_2_O_2_ to halt endogenous peroxidase activity. Non-specific binding sites were halted with 5% bovine serum in PBS. Rabbit polyclonal primary antibodies (ab28340, Abcam, USA, 1:1000 dilution) were utilized to identify DPP4. An EnVision Detection kit (GK500705: Gene Tech, China) was used to visualize antigens. This was followed by hematoxylin for 5 min. Negative control slides that did not have primary antibodies were used in all of the assays. IOD/µm^2^ was used for quantitative evaluation of DPP4 as previously reported (22). In brief, a Leica DM IRE2 microscope (Leica Microsystems Imaging Solutions Ltd., UK) that was connected to a Leica CCD camera DFC420 was used as the imaging system. Representative fields were photographed with high-powered magnification (×100) using Leica QWin Plus v3 software. The area (μm^2^) and IOD of each image were quantified and counted with Image-Pro Plus v6.0 software (Media Cybernetics, Inc., USA).

### PDX model

Data of tumor growth were adapted from previous studies (23). Briefly, surgically resected primary tumor tissues (designated as PA) were subcutaneously implanted in female BALB/c nude mice (Shanghai SLAC Laboratory Animal Co., Ltd., China). The first generation of xenografts was set as P0. Tumor volume was quantified as 0.5 × length × width^2^. Volumes were plotted against time to create growth curves. We adapted 29 cases of PA tissues and constructed a tissue microarray, and DPP4 expression was performed by immunohistochemical analysis. Tumors were measured every 2 weeks on the first 30 days, and once a week after 30 days. Because the growth rate of the tumor varies greatly, the time-point of tumor rapid growth was taken as the starting point, and the tumor volume from that point was compared with the tumor volume at the starting point (ratio = tumor volume of next week / tumor volume at the starting point).

### Statistical analysis

Chi-squared analyses and survival analyses were conducted with SPSS standard version 13.0 (SPSS, USA). Histograms and growth curves were created with GraphPad Prism 6.02 (USA). The best cut-off point for DPP4 expression was found with X-tile analysis (software version 3.6.1, Yale University School of Medicine, USA) (24). A difference was established as being significant if the P value from a two-tailed test was less than 0.05.

## Results

### DPP4 protein was decreased in HCC tissues

**Figure 1:**
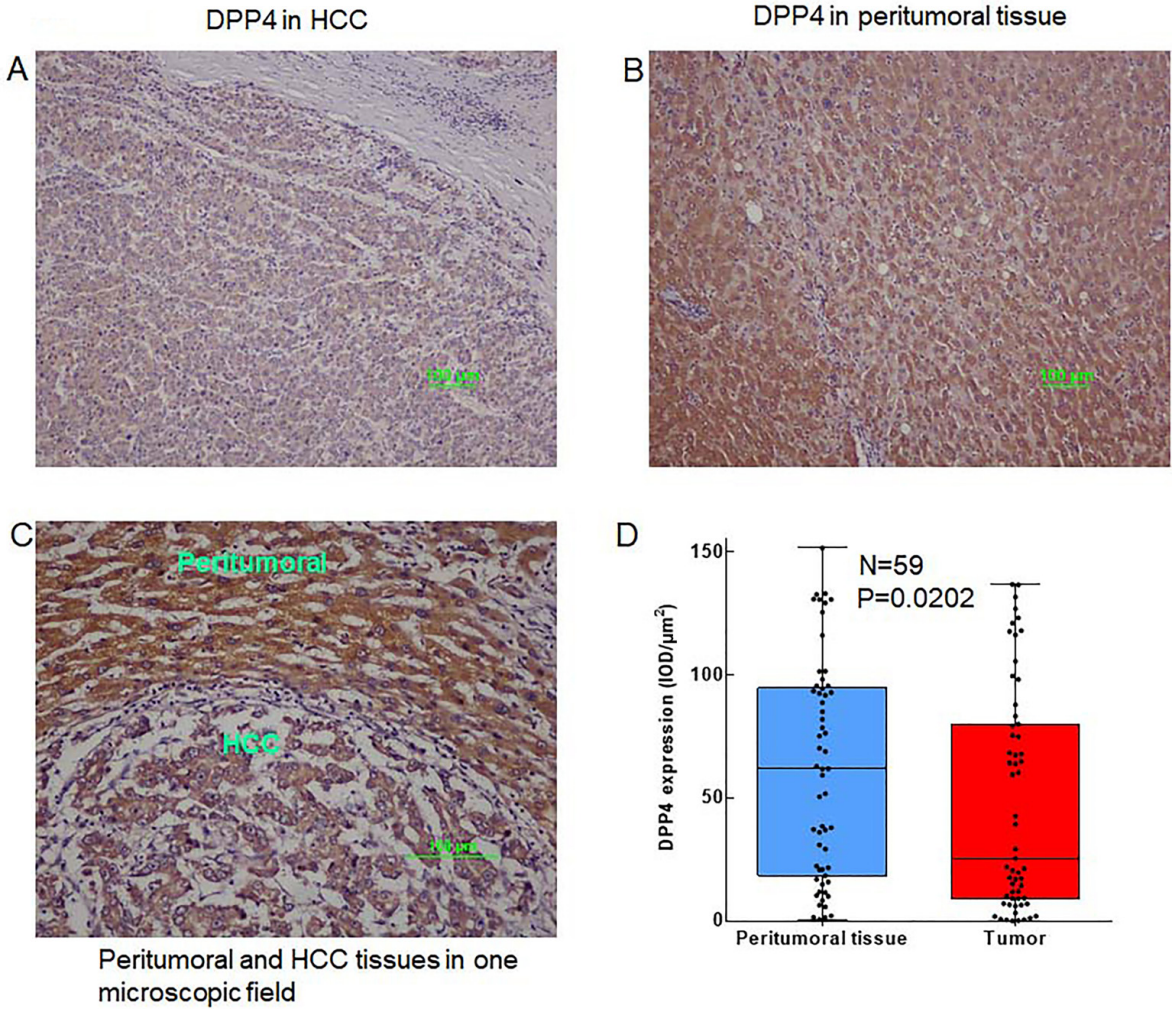
Expression of dipeptidyl peptidase 4 (DPP4) in (**A**) hepatocellular carcinoma (HCC) tissue, (**B**) paired peri-tumoral liver tissue, and (**C**) HCC tissues and peri-tumoral tissues in one microscopic field (×100, scale bar 100 μm). **D**, Box plot showing the expression level (IOD/µm^2^) of DPP4 in HCC tissue (n=59). Data are reported as median and interquartile range. P=0.0202, chi-squared test.

Tissue microarray-based immunohistochemical analysis for detecting DPP4 expression showed that DPP4 was predominantly decreased in HCC tissues (Figure 1A) compared with that of paired peri-tumoral tissues (Figure 1B). Figure 1C, that shows HCC tissues and peri-tumoral tissues in one microscopic field, clearly illustrated this phenomenon, and significant statistical differences were observed using 59 paired HCC and peri-tumoral liver tissues (Figure 1D) (P=0.0202).

### Correlation among DPP4 expression and clinicopathological features

The best cut-off point was acquired from X-tile software evaluations. A total of 327 patients were placed into the low DPP4 expression group (n=246) and high DPP4 expression group (n=81). Chi-squared analysis of clinical-pathological variables and DPP4 expression in 327 patients revealed that DPP4 expression was closely correlated with TNM stage (P=0.038), tumor number (P=0.035), and vascular invasion (P=0.024), while DPP4 expression was not correlated with age, sex, HBsAg, serum AFP, liver cirrhosis, Child-Pugh class, tumor differentiation, or tumor size (Table 1).

**Table 1:** Relationship of DPP4 expression and clinicopathological features in hepatocellular carcinoma patients.

Variable	DPP4 expression		
	Low (n,%)	High (n,%)	P
Gender			0.137
Male	219 (66.9%)	67 (20.5%)	
Female	27 (8.3%)	14 (4.3%)	
Age (years)			0.976
≤50	121 (37.1%)	40 (12.2%)	
>50	125 (38.2%)	41 (12.5%)	
HBsAg			0.248
Negative	38 (11.6%)	17 (5.2%)	
Positive	208 (63.6%)	64 (19.6%)	
Serum AFP			0.082
≤20 ng/mL	83 (25.4%)	36 (11.0%)	
>20 ng/mL	163 (49.8%)	45 (13.8%)	
Liver cirrhosis			0.172
No	74 (22.6%)	18 (5.5%)	
Yes	172 (52.6%)	63 (19.3%)	
TNM			**0.038**
I	73 (22.3%)	35 (10.7%)	
II	127 (38.8%)	38 (11.6%)	
III-IV	46 (14.1%)	8 (2.5%)	
Child-pugh class			0.254
A	226 (69.1%)	71 (21.7%)	
B	20 (6.1%)	10 (3.1%)	
Tumor size			0.680
≤5 cm	115 (35.2%)	40 (12.2%)	
>5 cm	131 (40.1%)	41 (12.5%)	
Tumor number			**0.035**
Single	185 (56.6%)	70 (21.4%)	
Multiple	61 (18.6%)	11 (3.4%)	
Tumor differentiation			0.407
High	21 (6.4%)	6 (1.8%)	
Moderate	210 (64.3%)	73 (22.3%)	
Poor	15 (4.6%)	2 (0.6%)	
Vascular invasion			**0.024**
No	84 (25.7%)	39 (11.9%)	
Yes	162 (49.6%)	42 (12.8%)	

DPP4: dipeptidyl peptidase 4; HbsAg: hepatitis B surface antigen; AFP: alpha fetoprotein; TNM: tumor-node-metastasis. Chi-squared test was used for statistical analyses. Bold type indicates statistically significant differences.

### Low expression of DPP4 predicted malignancy and short overall survival for postoperative HCC patients

**Figure 2:**
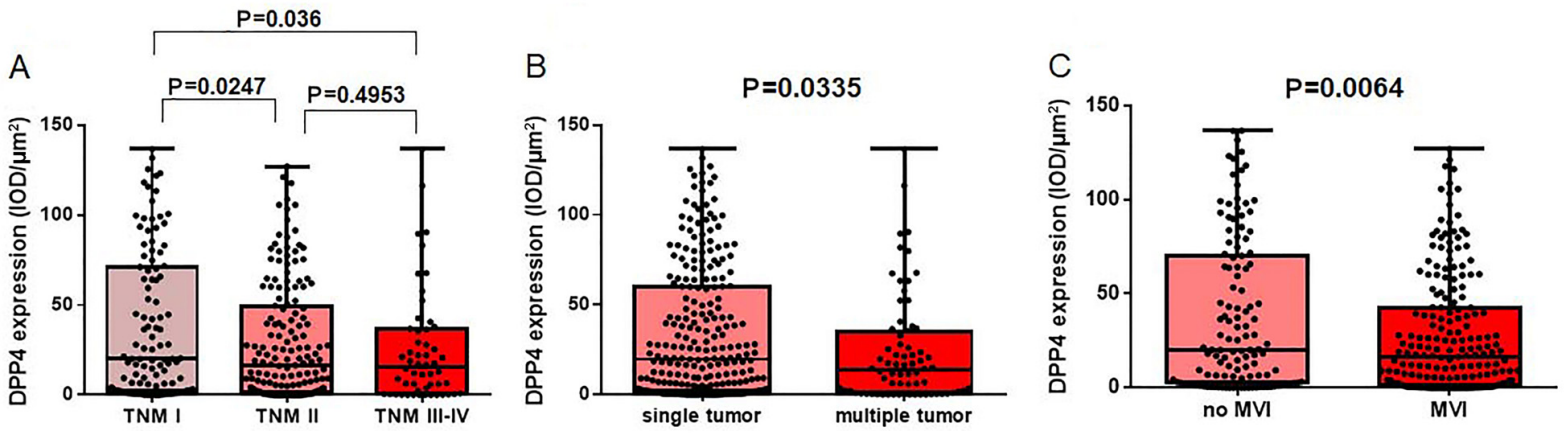
Expression of dipeptidyl peptidase 4 (DPP4) in (**A**) subgroups of TNM stage, (**B**) tumor number, and (**C**) microvascular invasion of tissues (MVI) of patients with hepatocellular carcinoma (HCC). Data are reported as median and interquartile range (chi-squared test).

**Figure 3:**
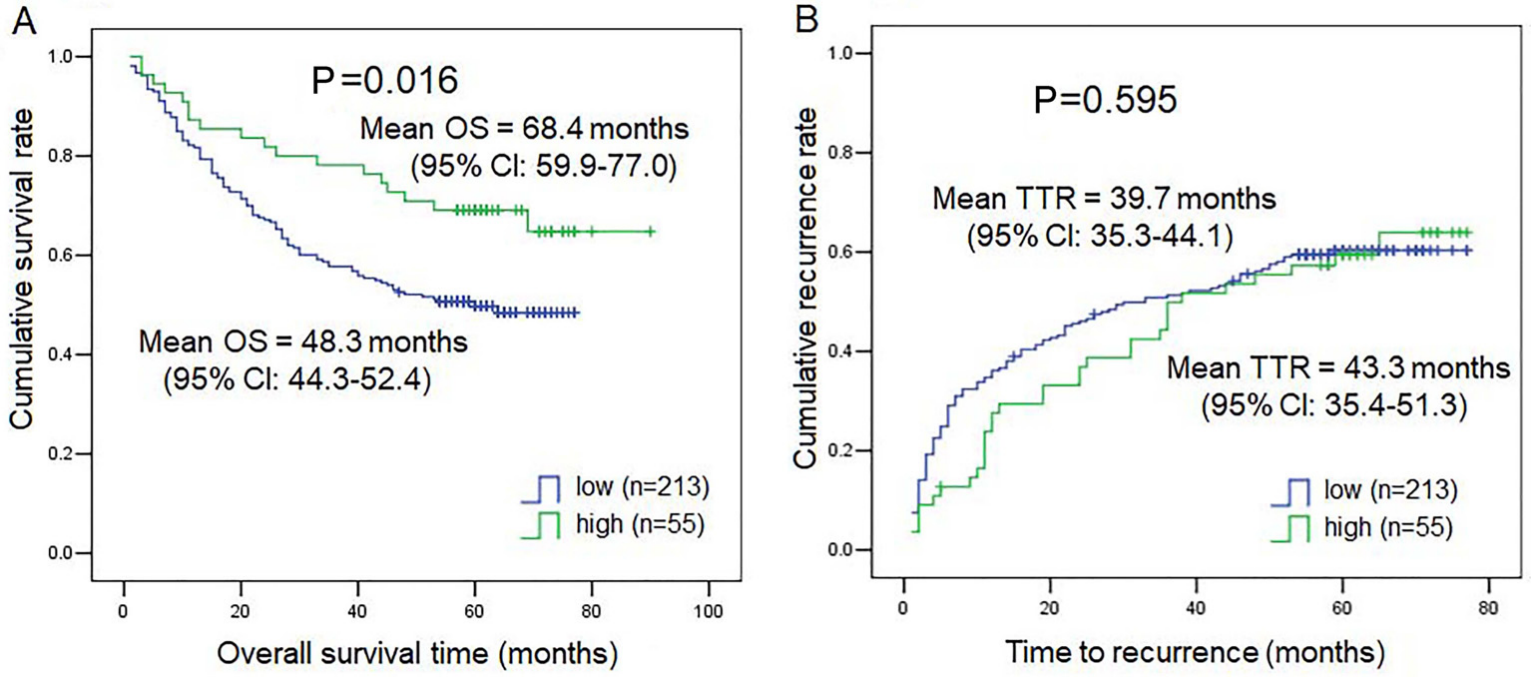
Kaplan-Meier curves for (**A**) overall survival (OS) and (**B**) time to recurrence (TTR) in patients with hepatocellular carcinoma (HCC) (268 cases) expressing low or high levels of dipeptidyl peptidase 4 (DPP4).

As shown in Table 1, TNM stage, tumor number, and vascular invasion correlated with DPP4 expression. Therefore, we further explored DPP4 expression in subgroups of TNM stage, tumor number, and vascular invasion. As shown in Figure 2A, DPP4 expression was much lower in the HCC tissues from patients with TNM stages II and III-V than in those from HCC patients with TNM I (P=0.0247 for TNM I *vs* TNM II, P=0.036 for TNM I *vs* TNM III-IV). Similarly, DPP4 expression was much lower in the HCC tissues from patients with multiple tumors (Figure 2B) and the presence of microvascular invasion (Figure 2C) compared to patients with a single tumor (P=0.0335) and no microvascular invasion (P=0.0064). Also, we compared time to recurrence and OS of 276 HCC patients in the low DPP4 expression group and the high DPP4 expression group. Subjects with low DPP4 expression in HCC tissues had a much lower mean survival time than those with high DPP4 expression (P=0.016, low DPP4=48.3 months *vs* high DPP4=68.4 months) (Figure 3A), while DPP4 was not associated with time-to-recurrence (Figure 3B) (P=0.595). Our results suggested an important role for DPP4 in the clinical behavior of HCC. Furthermore, the COX multivariate regression revealed that DPP4 was not an independent prognostic factor, but COX univariate regression analysis showed that DPP4 still had prognostic value for OS (P=0.019) (Supplementary Table S1).

### Low expression of DPP4 indicated higher aggressiveness in PDX model

**Figure 4:**
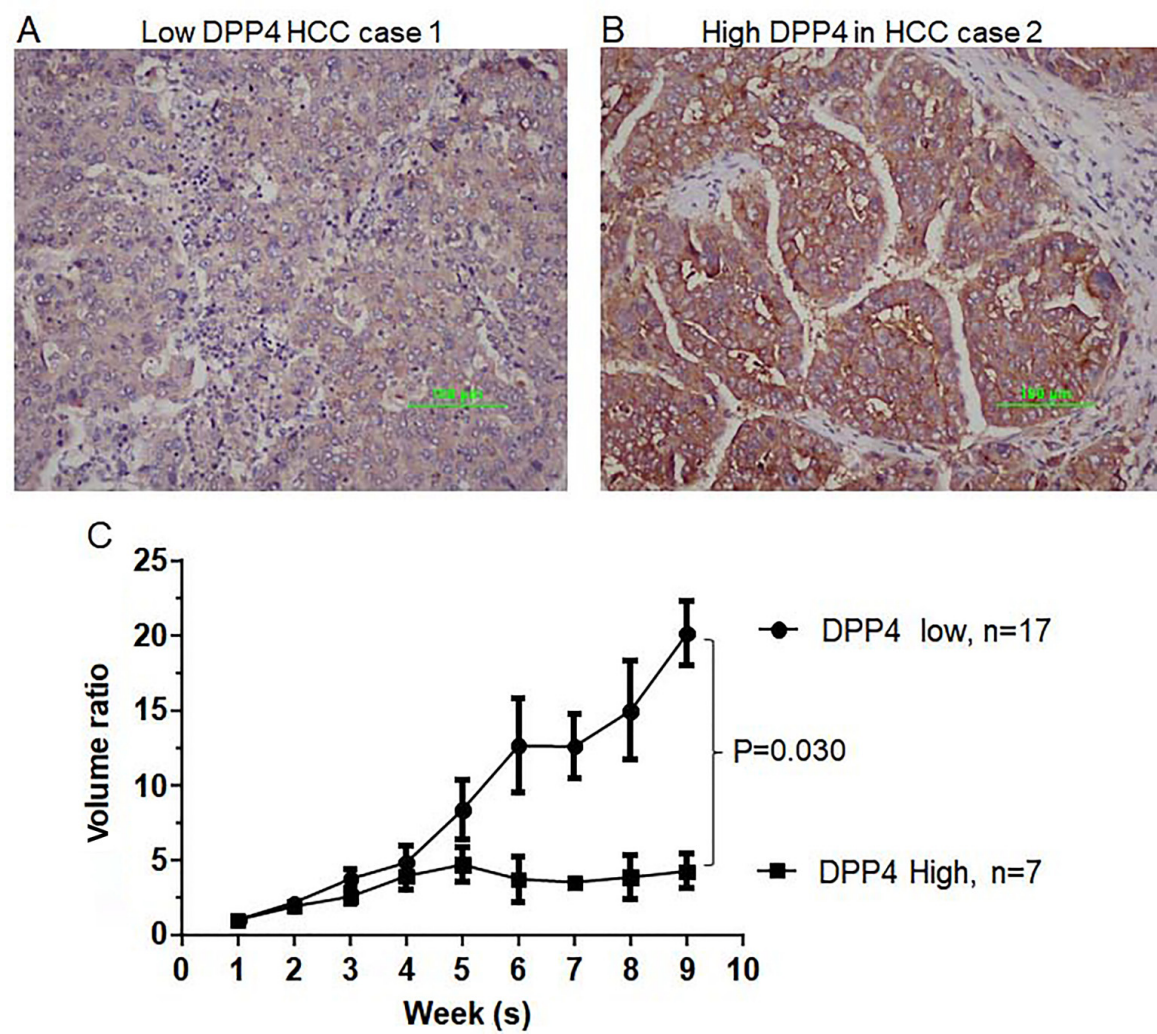
Photomicrographs of hepatocellular carcinoma (HCC) tissues with (**A**) low and (**B**) high expression of dipeptidyl peptidase 4 (DPP4) (×100, scale bar 100 μm). Tumor growth curves of HCC patient-derived xenograft models for low and high DPP4 expression groups (**C**). Data are reported as mean±SD.

As shown in Figure 4, PDX HCC tissues with low DPP4 expression (n=17) had more rapid tumor growth than PDX HCC tissues with high DPP4 expression (n=7). These results suggested that DPP4 may act as a tumor suppressor gene for HCC.

## Discussion

DPP4, also referred to as CD26, is a transmembrane glycoprotein of 110 kDa MW that is expressed constitutively in a dimeric form (220 kDa) in different cell types (25,26). Currently, most studies have reported about the clinical significance of serum DPP4 or DPP4 enzymatic activity. The serum levels of DPP4 have been documented as a pivotal diagnostic or prognostic biomarker in a few types of tumors.

Although it has been reported that hepatocyte-secreted DPP4 in obesity encourages adipose inflammation and insulin resistance (27), and inhibition of dipeptidyl peptidase IV halts high-fat diet-induced liver cancer angiogenesis by downregulating chemokine ligand 2 (28), the DPP4 expression level in HCC tissue has been unclear. It is still unknown whether DPP4 has a part in HCC as an oncogene or tumor suppressor gene.

In the current investigation, we demonstrated that the expression of DPP4 protein was lower in HCC tissues in contrast to peri-tumoral liver tissues. Low DPP4 expression in HCC tissues may indicate worse OS rates. Furthermore, DPP4 expression had an inverse correlation with the aggressiveness of HCC, such as the TNM state, tumor number, and microvascular invasion (Figure 2). These findings are similar to those reported previously that DPP4 expression was a significant factor in endometrial adenocarcinoma and it had an inverse correlation with tumor grade (18). More importantly, we observed that HCC tissues with low DPP4 expression had more rapid tumor growth than HCC tissues with high DPP4 expression using the PDX model, and this result indicated that DPP4 may act as a tumor suppressor gene for HCC.

Davoodi et al. reported that glypican-3 binds to and inhibits the dipeptidyl peptidase activity of DPP4 in the Simpson-Golabi-Behmel syndrome (29). Glypican-3 is a unique protein specifically expressed in HCC cells and the high level of glypican-3 expression may indicate tumor malignancy and predict the patient's prognosis (30,31). The relationship of DPP4 and glypican-3 expression in HCC, the mechanism of DPP4 down-regulation in HCC, and whether glypican-3 participates in its expression process should be explored by further research.

It was reported that DPP4 plays crucial roles in the development of various chronic liver diseases (32), and high DPP4 expression in HCC specimens was positively associated with poorer prognosis in HCC patients (n=41) (33). However, the present study showed that DPP4 decreased in HCC and low DPP4 expression positively correlated with poor prognosis of HCC patients (n=268). In addition, McCaughan et al. (34) reported that DPP4 is markedly reduced at the protein and mRNA levels in rat hepatoma cells compared with rat hepatocytes. Based on the results from our larger sample size and direct comparison of HCC tissue and paired peri-tumoral tissues, we suggest that further multicenter and larger studies should be performed to confirm the findings.

It is noteworthy that DPP4 mRNA expression levels were slightly greater in HCC tissues in contrast to paired liver tissues (Supplementary Figure S1A), and DPP4 mRNA expression was not related with OS of HCC patients (Supplementary Figure S1B). However, as shown in the present study, the DPP4 protein expression level and prognostic role significantly disagreed with the mRNA level. We speculate that this might be due to different cohorts of study population and to post-transcriptional modification and post-translational modifications. In general, the results of protein levels are more important, and the final biological effects are usually due to protein action.

In conclusion, expression of DPP4 protein decreased in HCC tissues and may indicate worse OS of HCC patients after surgery; DPP4 may act as a tumor suppressor gene for HCC.

## Supplementary Material

Click here to view [pdf].
